# Porcine placenta hydrolysates enhance osteoblast differentiation through their antioxidant activity and effects on ER stress

**DOI:** 10.1186/s12906-016-1274-y

**Published:** 2016-08-17

**Authors:** Hwa-Young Lee, Han-Jung Chae, Sun-Young Park, Jong-Hyun Kim

**Affiliations:** 1 Department of Pharmacology and Institute of Drug Development, Chonbuk National University, Jeonju, 560-182 Republic of Korea; 2 CODEBIO CO., LTD, Busong 1gil 62, Jiksan-eup, Seobuk-gu, Cheonan, Chungnam, 331-815 Republic of Korea; 3 Department of Obstetrics and Gynecology, Institute for Medical Sciences, Chonbuk National University Medical School, Jeonju, 560-182 Republic of Korea

**Keywords:** Osteoporosis, Oxidative damage, Alkaline phosphatase, Apoptosis, ROS

## Abstract

**Background:**

Osteoporosis is a disease characterized by decreased bone strength, decreased bone mass, and bone deterioration. Oxidative damage is an important contributor to functional changes in the development of osteoporosis. Here we found that porcine placenta hydrolysates (PPHs) protect MC3T3-E1 osteoblastic cells against hydrogen peroxide (H_2_O_2_)-induced oxidative damage.

**Methods:**

In vitro cell viability was determined using trypan blue dye exclusion. ER stress and apoptosis were evaluated using immunoblotting and a commercially available caspase kit. ALP, osteocalcin, Runx2, and osterix expression levels were evaluated by RT-PCR using isolated RNA. ROS, NADPH oxidase, and SOD activity levels were also measured.

**Results:**

We investigated the mechanisms underlying PPH-mediated inhibition of H_2_O_2_-induced ER stress and ROS production. PPHs also regulated osteoblast differentiation via the upregulation of alkaline phosphatase (ALP) expression in MC3T3-E1 osteoblastic cells. Also, treatment with PPHs enhanced the transcription of osteocalcin, Runx2, and osterix. These effects were all associated with the antioxidant actions of PPHs. Moreover, PPHs reversed the decrease in SOD activity, decreased ROS release, and inhibited NADPH oxidase activity in H_2_O_2_-treated MC3T3-E1 osteoblastic cells.

**Conclusions:**

PPHs protect cells against H_2_O_2_-induced cell damage when ER stress is involved. In addition, PPHs enhance osteoblast differentiation. This enhancement likely explains the regulatory effect of PPHs on bone metabolism disturbances, i.e. PPHs control ER stress and the related ROS production in osteoblasts.

## Background

Osteoporosis is characterized by decreased bone strength, decreased bone mass, and bone tissue deterioration. An imbalance between bone resorption and bone formation is the dominant mechanism causing osteoporosis [1, 2]. Since new bone formation primarily depends on osteoblasts, factors that disturb their bone-forming characteristics can lead to bone formation defects or related pathological conditions. Osteoblasts are secretory cells with well-developed endoplasmic reticulum (ER) cristae, in which many secretory proteins are folded and then secreted. The balance of osteoblasts and osteoclastic cells is carefully controlled to maintain bone and the related endocrinology homeostasis [1, 3, 4]. ROS are one of the key factors responsible for dysregulation of bone maintenance, especially osteoblast physiology. Osteoblasts are coated with high levels of secreted proteins. When these cells do not work effectively, ROS affect the secretory function of the osteoblasts, resulting in detrimental signaling to osteoclasts. Furthermore, during severe pathologic stress, osteoblasts undergo apoptosis. This also disturbs the balance between osteoblasts and osteoclasts and ultimately leads to bone resorption and related disease conditions [5].

The ER plays a major role in controlling protein folding and secretion in cells. Various acute and chronic conditions, including protein misfolding and Ca^2+^ disturbances, can alter ER function and lead to ER stress [6–8]. Furthermore, ER stress has been reported to contribute to several diseases, including many bone diseases [9, 10]. Osteoblast apoptosis associated with ER stress is one of the predominant mechanisms of osteoporosis pathogenesis [11–14]. In stressed osteoblasts, endocrine function, including the production of bone formation hormones (e.g., osteopontin and osteocalcin), is impaired [15, 16]. Under stress conditions, ROS (e.g. H_2_O_2_) have been identified as key detrimental messengers. Moreover, ROS have also been associated with ER stress in other pathological conditions [17–19]. Numerous studies have evaluated the role of ROS and ER stress in bone development [3, 20–22]. Therefore, the identification of interventions that can control ROS-associated ER stress is important for preventing and treating bone disease.

The placenta is an organ found exclusively in women during pregnancy that supplies nutrients and oxygen to the developing fetus. The nutritional substances and vitamins therein can be extracted in the form of porcine placenta hydrolysates (PPHs). PPHs contain beneficial bioactive components that inhibit aging, sunburns, and oxidation [23, 24]. PPHs have also been used for wound healing in Korean folk medicine [25, 26] and have been demonstrated to exert immunomodulatory effects [27, 28]. However, the effect of PPHs on bone formation remains unclear. To determine the role of PPHs in the endocrine system, it is important to determine the effects of PPHs on osteoblasts, a representative endocrine cell associated with stress conditions. The aim of our study was to evaluate the effects of PPHs on H_2_O_2_-induced oxidative damage and the related ER stress in MC3T3-E1 osteoblastic cells. We found that PPHs regulate oxidative ER stress and the associated activation of antioxidants, thus enhancing differentiation of MC3T3-E1 osteoblastic cells in the presence of H_2_O_2_.

## Methods

### Materials

PPHs were purchased from Codebio Inc. (Cheonan, Republic of Korea). Hydrogen peroxide and thapsigargin were obtained from Sigma Chemical Company (St. Louis, MO, USA). Caspase-3 and -12 activity kits were obtained from BioVision (Mountain View, CA, USA). All other reagents used were of analytical grade and were generally obtained from Sigma.

### Extraction and quantitation of PPHs using HPLC-DAD

PPHs were purchased from Codebio Inc. (Cheonan, Republic of Korea). Placentas were thawed using a defroster and then washed with saline at 16 °C to remove blood and cords. Placentas were hydrolyzed using papain, bromelain, pronase, and Alcalase at 70 ± 0.1 °C for 2 days (pH 4.5–6.0). Next, hydrolytic enzymes were inactivated at 100 ± 0.1 °C for 30 min and the resultant hydrolysates were filtered. The lipids were then removed by mixing with calcium and phosphate salts and by filtering the insoluble particulate matter. Finally, the hydrolysates were adjusted to pH 7.0 ± 0.2 using calcium and phosphate salts. Hydrolysates were mixed with AccQ-Fluor buffer and Acc-Fluor reagent (Waters Corporation, Milford, MA, USA), after which the mixture was heated at 80 °C. An aliquot of the sample was filtered and injected into an AccQ-Tag column (150 × 2.1 mm, 3 μm particle size; Waters Corporation, Milford, MA, USA) in an HPLC instrument for chromatographic separation. A gradient mixture of AccQ-Tag Eluent (A) and acetonitrile (B) was used as the mobile phase at a flow rate of 1 mL/min at 30 °C. The initial eluent was 100 % A, which linearly decreased to 67 % A over 33 min. The eluent was switched to 100 % B for 3 min and then switched back to 100 % A for 26 min. Amino acids were detected using a fluorescence detector at excitation and emission wavelengths of 250 and 395 nm, respectively.

### Cell culture and viability analysis

The murine calvaria-derived MC3T3-E1 osteoblast-like cell line (4 to 10 passages) was purchased from the American Type Culture Collection (ATCC; Manassas, VA, USA). Cells were seeded at 1 × 10^5^ cells/mL and maintained in minimum essential medium (α-MEM) supplemented with 10 % fetal bovine serum (FBS) and penicillin-streptomycin (Invitrogen). Cells were incubated at 37 °C in a 95 % air/5 % CO_2_ atm. Cell viability was detected using a trypan blue exclusion assay. Briefly, MC3T3-E1 cells (5 × 10^4^ cells/well) were incubated in 12-well plates overnight and treated for 24 h with drugs in medium containing 10 % serum. Cells were washed with sterile phosphate-buffered saline (PBS), treated with 0.25 % trypsin-EDTA (GIBCO BRL), and harvested. Cells were diluted in 0.1 % trypan blue (GIBCO BRL) and then counted under a light microscope.

### Immunoblotting

For immunoblotting, MC3T3-E1 osteoblastic cells were lysed with extraction buffer. Proteins in the resultant lysates (40 μg) were resolved on a polyacrylamide gel and transferred to a nitrocellulose membrane. The blots were probed overnight at 4 °C with primary antibodies, washed, and probed again with species-specific secondary antibodies coupled to horseradish peroxidase (GE Healthcare, Piscataway, NJ, USA). Chemiluminescence reagents (GE Healthcare) were used for detection. Primary antibodies consisted of rabbit anti-GADD153/C/EBP homologous protein (CHOP), rabbit-anti-PERK, rat anti-GRP78, rabbit anti-ATF6α, mouse anti-eIF2α, mouse anti-β-actin (Santa Cruz Biotechnologies, Inc., Santa Cruz, CA, USA), rabbit anti-IRE1α, and rabbit anti-p-eIF2 (Cell Signaling Technologies, Inc., Danvers, MA, USA).

### Measurement of caspase-3 activity

To analyze caspase-3 activity, pellets were resuspended in extraction buffer [25 mM HEPES (pH 7.4), 0.1 % Triton X-l00, 10 % glycerol, 5 mM DTT] and spun by centrifugation at 13,000 rpm at 4 °C for 30 min. Soluble protein (40 μg) was mixed with 100 μM caspase-3-specific substrate Ac-DEVD-AFC (Sigma-Aldrich) and incubated at 37 °C. Caspase-3 activity was analyzed by monitoring fluorogenic AFC release at 37 °C. Substrate cleavage was monitored at 405 nm using a SPECTRAmax 340 microplate reader and analyzed using SOFTmax PRO software (Molecular Devices, Sunnyvale, CA, USA).

### Measurement of caspase-12 activity

To analyze caspase-12 activity, pellets were measured by detecting free AFC cleavage by caspase-12-specific substrates. These experiments were performed with a caspase-12 Assay Kit (Biovision, San Francisco, CA, USA). After the lysates were incubated with ATAD-AFC for 2 h at 37 °C, the absorbance of each sample was analyzed at 505 nm.

### Measurement of alkaline phosphatase activity

To analyze ALP activity, cells were treated with Krebs (control), PPHs (100 μg/mL), H_2_O_2_ (400 μM), and in the presence or absence of 100 μg/mL PPHs. After treatment for 4 h, cells were lysed. The amount of ALP activity and the protein concentration were measured in each supernatant using an ALP activity assay kit (Cell Biolabs, San Diego, CA, USA).

### RT-PCR

The mRNA levels of ALP, osteocalcin, Runx2, osterix, and GAPDH were determined using a PrimeScript™ RT reagent Kit (TaKaRa Bioscience, Kyoto, Japan). The sequences of the primers used for RT-PCR were as follows: ALP, forward primer: 5′-CCATGGTAGATTACGCTCACA-3′, reverse primer: 5′-ATGGAGGATTCCAGATACAGG-3′; osteocalcin, forward primer: 5′-AGCTATCAGACCAGTATGGCT-3′, reverse primer: 5′-TTTTGGAGCTGCTGTGACATC-3′; Runx2, forward primer: 5′-CTCAGTGATTTAGGGCGCATT-3′, reverse primer: 5′-AGGGGTAAGACTGGTCATAGG-3′; Osterix, forward primer: 5′- CGGGTCAGGTACAGTG-3′, reverse primer: 5′- ACCATGACGACAAGGG-3′; and GAPDH, forward primer: 5′-ATCACCATCTTCCAGGAG-3′, reverse primer: 5′-ATGGACTGTGGTCATGAG-3′. Reverse transcription was performed by incubating the reactions at 37 °C for 15 min and then at 85 °C for 5 s. For polymerase chain reaction amplification, an initial denaturation step was performed at 94 °C for 3 min, followed by annealing at 55 °C for 20 s and elongation at 72 °C for 45 s. In total, 35 cycles were performed.

### NADPH oxidase activity assay

Cells were seeded in six-well plates and cultured for 48 h. Next, the cells were treated with 100 μM H_2_O_2_ for 6 h in the presence or absence of 100 μg/mL PPHs. NADPH oxidase activity was determined based on superoxide-induced lucigenin photoemission as described by Rao and Maddala et al. [29]. Enzymatic assays were performed in a final volume of 0.2 ml containing 50 mM phosphate buffer (pH 7.0), 1 mM EGTA, 150 mM sucrose, 0.5 mM lucigenin, 0.1 mM NADPH, and cell lysis solution. Enzymatic reactions were initiated by the addition of lucigenin. Photoemission, expressed as relative light units, was measured every minute for 10 min using a luminometer. Assays were performed in the dark at room temperature with all appropriate controls.

### Superoxide dismutase (SOD) activity assay

Cells were seeded in six-well plates and cultured for 48 h. The cells were then treated with 100 μM H_2_O_2_ for 6 h in the presence or absence of 100 μg/mL PPHs. Next, cells were harvested and the level of SOD activity was determined using a SOD assay kit (k335-100, Biovision) according to the manufacturer’s instructions.

### DCFDA assay (ROS production)

The cellular ROS level was measured by following the protocol described by Badham et al. [30]. Briefly, cells were treated with 100 μM H_2_O_2_ at 37 °C in the presence or absence of 100 μg/mL PPHs for 6 h. Next, cells were incubated with 10 μM 2′, 7′-dichlorofluorescein diacetate (DCFDA) at 37 °C for an additional 30 min. The fluorescence intensity of 2′,7′-dichlorofluorescein, a product of the reaction between DCFDA and cellular ROS, was analyzed using a fluorescence reader (SpectraMax 190, Molecular Devices, Sunnyvale, CA, USA). Data were normalized to the H_2_O_2_ absorbance values.

### Statistical analysis

Results are presented as means ± standard errors of the mean (SEMs) for multiple wells tested in at least three separate experiments. MicroCal Origin software (Northampton, MA, USA) was used for statistical calculations. Differences were tested for significance using one-way analysis of variance (ANOVA) with Duncan’s multiple range test.

## Results

### PPHs protect against H_2_O_2_-induced cell death in MC3T3-E1 osteoblastic cells

**Fig. 1:**
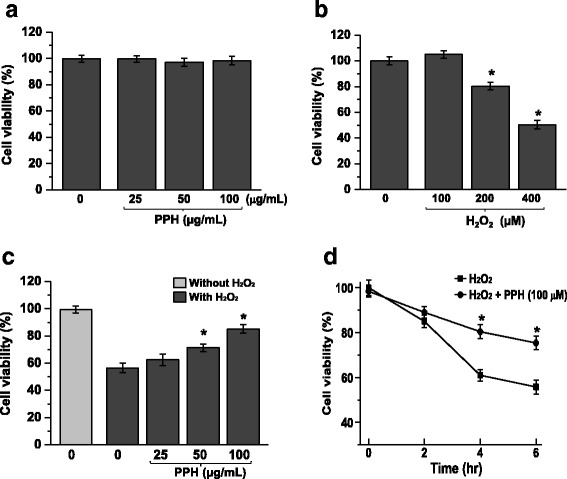
PPHs regulate H_2_O_2_-induced cell death in MC3T3-E1 osteoblastic cells. **a** To test the cell viability of PPHs in MC3T3-E1 cells, cells were treated with 0, 25, 50, or 100 μg/mL PPHs only for 6 h. Cell viability was estimated via the trypan blue exclusion test. Untreated cells were used as the control. **b** To test the cell viability of H_2_O_2_ in MC3T3-E1 cells, cells were treated with 0, 100, 200, or 400 μM H_2_O_2_ only for 6 h. Cell viability was estimated via the trypan blue exclusion test. Untreated cells were used as the control. **c** Cells were treated with 0, 25, 50, or 100 μg/mL PPHs in the presence or absence of 400 μM H_2_O_2_ for 6 h, and cell survival was assessed. **d** Cells were exposed to 400 μM H_2_O_2_ in the presence or absence of 100 μg/mL PPHs for 0, 2, 4, or 6 h, after which cell viability was assessed. ^*^ *p* < 0.05 versus cells treated with H_2_O_2_ alone (*n* = 3). *PPHs* porcine placenta hydrolysates

We first determined if PPHs exert concentration-dependent effects on cell viability. Treatment with PPHs for 6 h at concentrations ranging from 25 to 100 μg/mL did not have any significant effect on cell survival (Fig. 1a). However, treatment of cells for 6 h with 100, 200, or 400 μM H_2_O_2_ markedly increased cell death in a concentration-dependent manner (Fig. 1b). Interestingly, treatment of cells with 400 μM H_2_O_2_ and 25, 50, or 100 μg/mL PPHs markedly affected cell survival in a concentration- and time-dependent manner (Fig. 1c and d). Moreover, treatment with 100 μg/mL PPHs for 30 min significantly protected cells against H_2_O_2_-induced (400 μM) cell damage. These findings suggest that PPHs significantly protect MC3T3-E1 osteoblastic cells.

### PPHs protect against H_2_O_2_-induced apoptosis in MC3T3-E1 osteoblastic cells

**Fig. 2:**
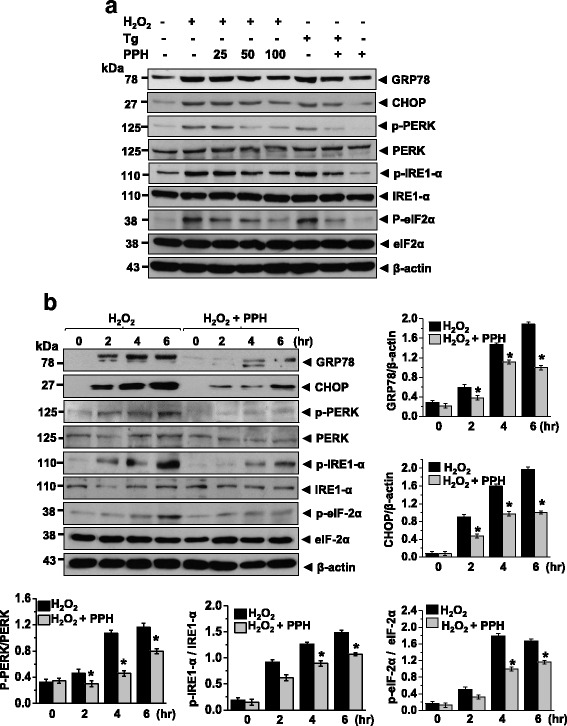
PPHs decrease the H_2_O_2_-induced ER stress response in MC3T3-E1 osteoblastic cells. **a** Cells were exposed to 400 μM H_2_O_2_ or 0.1 μM Tg and 0, 25, 50, or 100 μg/mL PPHs for 6 h. Immunoblotting was performed using the indicated antibodies. **b** Cells were exposed to 400 μM H_2_O_2_ in the presence or absence of 100 μg/mL PPHs for 0, 2, 4, or 6 h. Immunoblotting was performed using the indicated antibodies. Blots are representative of three independent experiments. ^*^ *p* < 0.05 versus cells treated with H_2_O_2_ alone (*n* = 3). *PPHs* porcine placenta hydrolysates, *Tg* thapsigargin

**Fig. 3:**
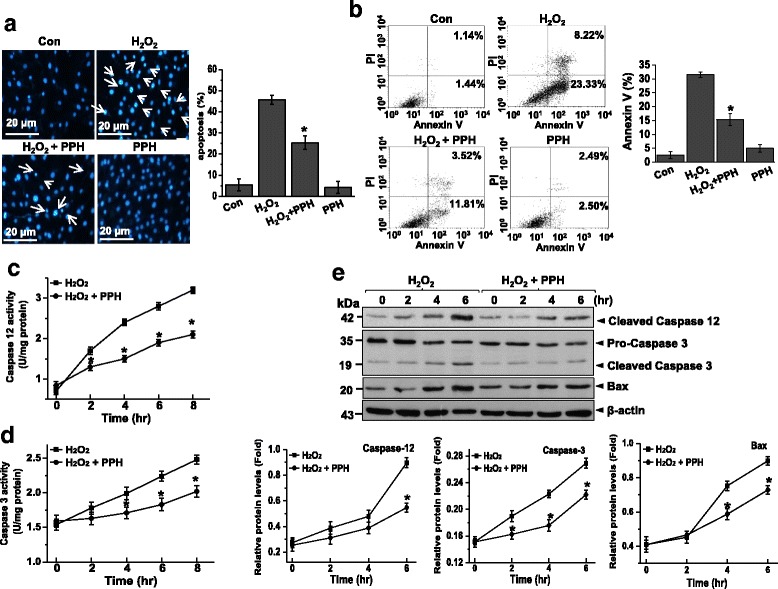
PPHs protect against H_2_O_2_-induced apoptosis in MC3T3-E1 osteoblastic cells. **a** Apoptosis was detected by Hoechst dye staining and quantified based on nuclear condensation or fragmentation (*right*). Representative pictures are shown from three independent experiments. Images were taken at a magnification of 400×. *Scale bar*, 20 μm. **b** Cells were stained with FITC-conjugated Annexin V and PI, followed by flow cytometric analysis. **c** Caspase-12 activity was analyzed in cells treated for 0, 12, 24, 36 or 48 h with 400 μM H_2_O_2_ in the presence or absence of 100 μg/mL PPHs. **d** Caspase-3 activity was measured in cells that were treated for 6 h with 400 μM H_2_O_2_ in the presence or absence of 100 μg/mL PPHs. **e** Cells were treated for 0, 2, 4, or 6 h with 400 μM H_2_O_2_ in the presence or absence of 100 μg/mL PPHs. Immunoblotting was performed using the indicated antibodies. Representative blots are shown from three independent experiments. *Bottom panel*, quantitative immunoblot data (*n* = 4). ^*^ *p* < 0.05 versus cells treated with H_2_O_2_ alone. *PPHs* porcine placenta hydrolysates

To evaluate whether PPHs could protect against ER stress in osteoblasts, cells were treated with 400 μM H_2_O_2_ for 6 h or 0.1 μM Tg (thapsigargin, a Ca^2+^-ATPase inhibitor) for 24 h to induce ER stress. To investigate the ER stress response, the levels of GRP78, CHOP, p-PERK, p-eIF2α, p-IRE1-α, and ATF6α were analyzed by immunoblotting. The levels of these proteins were all significantly increased in cells treated with 400 μM H_2_O_2_ or 0.1 μM Tg. However, treatment with PPHs inhibited this upregulation of GRP78, CHOP p-PERK, p-eIF2α, p-IRE1-α, and ATF6α (Fig. 2a and b). Moreover, PPHs inhibited H_2_O_2_-induced cell injury. Apoptosis levels were measured by flow cytometry and are expressed in units of mean fluorescence intensity. As shown in Fig. 3a-b, PPHs protected osteoblasts against H_2_O_2_-induced apoptosis. H_2_O_2_ treatment also induced nuclear condensation and fragmentation, characteristics of apoptosis. Pretreatment with PPHs markedly attenuated these effects (Fig. 3a). Moreover, the apoptosis level was 31.55 ± 1.02 % after H_2_O_2_ treatment for 6 h, whereas the apoptosis level in the PPHs group was 15.33 ± 2.1 % (Fig. 3b). These results confirm the protective effects of PPHs against H_2_O_2_-induced apoptosis in cells. Caspase-12 activation is associated with apoptosis [31]. Thus, we evaluated the effect of PPHs on caspase-12 activation. We found that caspase-12 activity was increased significantly after H_2_O_2_ treatment, whereas treatment with PPHs markedly reduced this H_2_O_2_-induced increase in caspase-12 activity (Fig. 3c). Next, we investigated caspase-3 activation. We found that H_2_O_2_ increased caspase-3 activation, whereas this increase was blocked by PPH treatment (Fig. 3d). Cells were exposed to H_2_O_2_ in the absence or presence of PPHs for 2, 4, and 6 h, after which the levels of apoptosis-related proteins were analyzed. H_2_O_2_ significantly increased the levels of caspase-12, caspase-3, and Bax in a time-dependent manner; in contrast, treatment with PPHs markedly reduced the protein levels of caspase-12, caspase-3, and Bax protein in MC3T3-E1 cells (Fig. 3e).

### PPHs attenuate H_2_O_2_-mediated inhibition of ALP activity

**Fig. 4:**
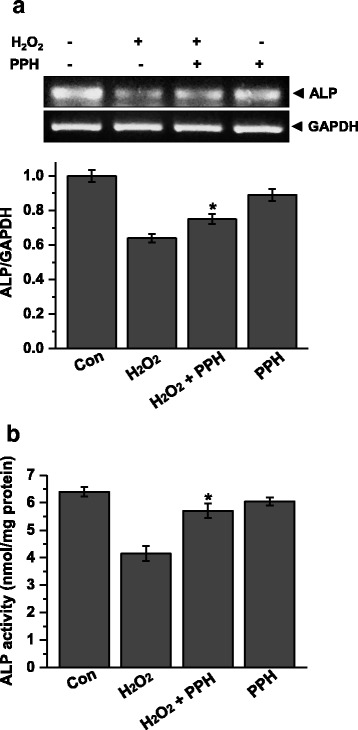
PPHs alleviate H_2_O_2_-mediated inhibition of ALP activity in MC3T3-E1 osteoblastic cells. **a** ALP mRNA levels in cells treated for 6 h with 400 μM H_2_O_2_ in the presence or absence of 100 μg/mL PPHs were analyzed by RT-PCR. Representative bands are shown from three independent experiments. Quantitative analysis of RNA expression was also performed. **b** ALP activity levels were analyzed in cells treated with 400 μM H_2_O_2_ in the presence or absence of 100 μg/mL PPHs (*n* = 3). ^*^ *p* < 0.05 versus cells treated with H_2_O_2_ alone. *PPHs* porcine placenta hydrolysates

To investigate the effect of PPHs on osteoblast differentiation, we investigated the mRNA level and activity of ALP in MC3T3-E1 cells. Treatment with 400 μM H_2_O_2_ for 6 h significantly decreased the mRNA level of ALP. However, treatment with PPHs markedly alleviated H_2_O_2_-mediated downregulation of ALP. Similarly, treatment for 6 h with 400 μM H_2_O_2_ markedly reduced ALP activity in cells. However, treatment with 100 μg/mL PPHs attenuated this effect of H_2_O_2_ (Fig. 4b).

### PPHs attenuate H_2_O_2_-induced downregulation of osteocalcin, Runx2, and osterix

**Fig. 5:**
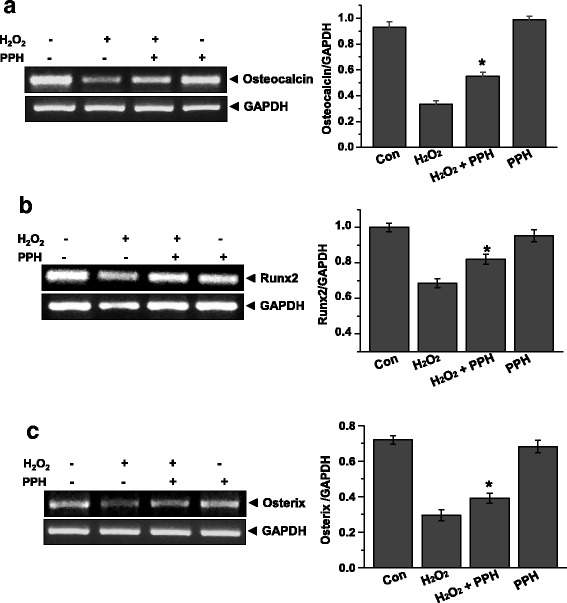
PPHs reverse the effects of H_2_O_2_ on the mRNA expression of osteocalcin, Runx2, and osterix in MC3T3-E1 osteoblastic cells. Osteocalcin (**a**), Runx2 (**b**), and Osterix (**c**) expression levels in cells treated for 6 h with 400 μM H_2_O_2_ in the presence or absence of 100 μg/mL PPHs were analyzed by RT-PCR. Representative bands are shown from three independent experiments. Quantitative analysis of RNA expression was also performed. ^*^ *p* < 0.05 versus cells treated with H_2_O_2_ alone. *PPHs* porcine placenta hydrolysates

To investigate the effects of PPHs on bone formation proteins, we investigated the mRNA levels of osteocalcin, Runx2 [32], and osterix [33]. Treatment for 6 h with 400 μM H_2_O_2_ markedly decreased the mRNA level of osteocalcin, whereas this change was markedly reversed by PPHs. Similarly, H_2_O_2_ markedly downregulated the mRNA levels of Runx2 and osterix (Fig. 5b and c). However, PPHs markedly attenuated this H_2_O_2_-mediated downregulation of Runx2 and osterix.

### PPHs affect ROS release, NADPH oxidase, and SOD activity in MC3T3-E1 osteoblastic cells

**Fig. 6:**
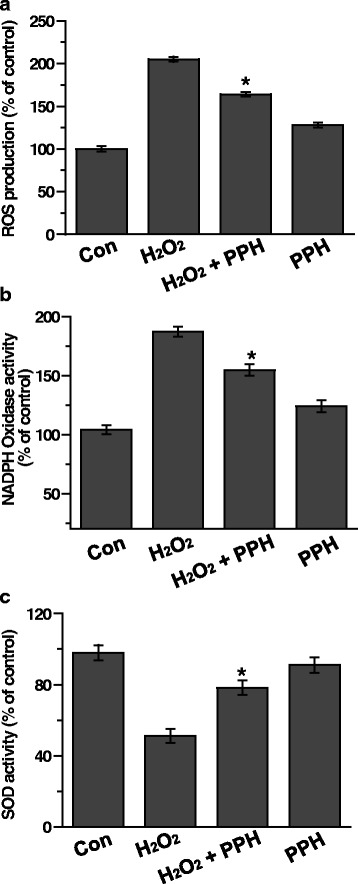
PPHs regulate ROS release, NADPH oxidase, and SOD activity in MC3T3-E1 osteoblastic cells. **a** ROS production was analyzed in cells treated for 6 h with 400 μM H_2_O_2_ in the presence or absence of 100 μg/mL PPHs by the DCF-DA assay. NADPH oxidase (**b**) and SOD activities (**c**) were assessed in cells treated for 6 h with 400 μM H_2_O_2_ in the presence or absence of 100 μg/mL PPHs. Representative bands are shown from three independent experiments. Quantitative analysis of RNA expression was also performed. ^*^ *p* < 0.05 versus cells treated with H_2_O_2_ alone. PPHs, porcine placenta hydrolysates

To evaluate the antioxidant effects of PPHs, we investigated the effect of PPHs on H_2_O_2_-mediated ROS production. We found that treatment with 400 μM H_2_O_2_ for 2, 4, or 6 h markedly increased ROS production. However, treatment with 100 μg/mL PPHs for 6 h markedly attenuated this H_2_O_2_–mediated increase in intracellular ROS (Fig. 6a). Next, we examined the effect of PPHs on the activity of NADPH oxidase, an enzyme that generates ROS. As shown in Fig. 6b, PPHs reduced H_2_O_2_-induced NADPH oxidase activity. These findings suggest that PPHs have antioxidant effects on osteoblasts. Next, we investigated the effect of PPHs on SOD activity. H_2_O_2_ treatment markedly inhibited SOD activity, whereas this effect was also alleviated by PPHs (Fig. 6c).

## Discussion

In this study, we found that PPHs protect MC3T3-E1 cells against H_2_O_2_-induced cell death and suppression of differentiation. H_2_O_2_-induced oxidative stress is known to lead to pathological ER stress. We found that PPHs, presumably via their antioxidant effects, protected MC3T3-E1 osteoblastic cells against H_2_O_2_-induced ER stress and ROS production. Moreover, PPHs significantly reversed H_2_O_2_-mediated downregulation of osteocalcin and two related proteins involved in bone formation, namely osterix and Runx2.

Reactive oxygen species (ROS) are generated by many environmental agents and have been implicated in the pathogenesis of various diseases, including osteoporosis [34]. Reduced bone formation by osteoblasts may cause osteoporosis to develop; moreover, the rate of osteoblast apoptosis regulates bone formation [1, 2, 35, 36]. ER stress has been implicated as an important factor in apoptosis due to various causes, including osteoblast apoptosis during the development of osteoporosis [11, 13, 14, 37]. The placenta has been studied for its potential to improve skin care, metabolic functions, and strengthen immune function [23]. However, it has remained unclear whether the placenta can protect osteoblasts from H_2_O_2_-induced ER stress and apoptosis. Here we evaluated the effects of PPHs on H_2_O_2_-induced ER stress and apoptosis in osteoblasts. ROS production is known to decrease bone formation by osteoblastic cells by suppressing the differentiation of osteoblast progenitors and the calcification process [38]. Therefore, decreased bone formation is strongly associated with enhanced oxidative stress and significantly decreased plasma levels of antioxidants in elderly persons with osteoporosis [39, 40]. In this study, H_2_O_2_ induced cell death, ER stress, and caspase-mediated apoptosis (Figs. 1, 2 and 3). However, PPH treatment prevented caspase activation, thus reducing the amount of apoptosis in response to ER stress. ER stress was recently reported to be involved in the induction of apoptosis during osteoporosis [11, 12, 14, 37]. ER stress is activated by the accumulation of unfolded proteins and alterations in calcium homeostasis [6–8]. Recent studies have also shown that ER stress-induced apoptosis of osteoblasts contributes to the development of osteoporosis [11–14, 37]. We found that ER stress was decreased in MC3T3-E1 cells (Fig. 2). However, treatment with PPHs increased osteoblast viability during periods of ER stress. Moreover, PPHs inhibited osteoblast apoptosis in response to ER stress. These results demonstrate that PPHs can protect osteoblasts from ER stress-induced apoptosis.

Caspases are the most prominent mediators of apoptosis. However, it is not clear if activated caspase-12 or caspase-3 plays an important role in H_2_O_2_-induced apoptosis [41]. In this study, we found that PPHs reversed the H_2_O_2_-induced activation of caspase-12 and caspase-3 (Fig. 3). These results demonstrate that PPHs can protect osteoblasts from caspase-mediated, H_2_O_2_-induced apoptosis.

H_2_O_2_-induced apoptosis is primary mediated by ROS production. We evaluated the protective effects of PPHs and found that they decreased H_2_O_2_-induced ROS release (Fig. 6). We also found that PPHs markedly inhibited NADPH oxidase activity. NADPH oxidase is important because it activates ROS-generating enzymes. PPHs also alleviated H_2_O_2_-mediated inhibition of SOD activity. The enhanced SOD activity observed upon treatment with PPHs may result in the scavenging of excessive superoxides derived from oxidative stress, thus ameliorating H_2_O_2_-induced cell death.

We also evaluated the effects of PPHs on osteoblastic differentiation. ALP is a primary marker of osteoblast differentiation. We found that H_2_O_2_ markedly downregulated ALP expression and that this effect was alleviated by PPHs (Fig. 4). Our data thus indicate that PPHs stimulate osteoblast differentiation. Furthermore, we also analyzed the mRNA levels of Runx2, osterix, and osteocalcin. Osteocalcin is a major protein produced by osteoblasts during bone formation. Osteocalcin expression is regulated by Runx2 and osterix levels [32, 42]. Therefore, we assessed the expression of osterix and Runx2 after treatment with PPHs and/or H_2_O_2_ (Fig. 5). We found that PPHs markedly reversed H_2_O_2_-mediated downregulation of osterix and Runx2 expression. These findings suggest that PPHs upregulate osteocalcin, osterix, and Runx2 expression.

It was beyond the scope of the present study to identify the active component(s) in PPHs. However, recent studies have suggested that dietary arginine and lysine may play an important role in bone development, growth, and modeling [43]. Moreover, arginine is thought to alleviate metabolic disturbances in calcium absorption, growth, dentition, and decalcification [44]. PPHs are known to contain high levels of arginine and other essential amino acids. Thus, our findings provide evidence that PPHs may have potential therapeutic value for treating bone metabolism disturbances in osteoblasts.

## Conclusions

Our findings suggest that PPHs, presumably via their antioxidant effects, protect MC3T3-E1 osteoblasts against H_2_O_2_-induced ER stress by suppressing ROS production. Furthermore, PPHs regulate oxidative stress-induced osteoporosis. Cumulatively, our findings suggest that PPHs may have potential therapeutic value for treating bone formation disturbances.

## Abbreviations

ALP: Alkaline Phosphatase
ER: Endoplasmic Reticulum
H_2_O_2_: Hydrogen Peroxide
PPHs: Porcine Placenta Hydrolysates
ROS: Reactive Oxygen Species
Tg: Thapsigargin
